# The F Breathing Circuit, a universal single-limb breathing circuit: brief historical perspective

**DOI:** 10.1007/s00540-019-02622-8

**Published:** 2019-03-11

**Authors:** Atsuo Fukunaga

**Affiliations:** Professor Emeritus, Department of Anesthesiology, Harbor-UCLA Medical Center, UCLA School of Medicine, 1000 West Carson Street, Torrance, CA 90509 USA

**Keywords:** Single-limb breathing circuit, Single-limb anesthetic circle circuit, Single-limb Mapleson rebreathing type circuit, Universal breathing circuit

## Abstract

This article presents a brief historical perspective of the F Breathing Circuit, a universal single-limb breathing circuit. The single-limb breathing circuit (tube within a tube configuration) comprises two tubes of sufficient size and compliance, so that either channel enables safe, unrestricted inspiration/expiration at all times in spontaneous and controlled ventilation. The single-limb circuit can function in various modes: as an anesthetic circle circuit as well as a Mapleson-type rebreathing circuit and as a non-rebreathing circuit (e.g., with ICU ventilators) in adult and pediatric patients. Therefore, it qualifies as a universal breathing circuit. Since its first introduction in Japan (1978), which was followed by further modifications and improvements, the circuit was made available in USA and worldwide.

## Anesthesia breathing circuit in modern anesthesia

This article presents the background and brief historical perspective of the F Breathing Circuit, the first universal single-limb breathing circuit (USLBC).

There have been two types of breathing systems or circuits used in anesthesia: (a) the circle circuit, which prevents carbon dioxide rebreathing by use of CO_2_ absorber while allowing partial rebreathing of other exhaled gases and (b) the Mapleson, semi-closed rebreathing circuit, which does not use CO_2_ absorption and the exhaled gases are washed out with high fresh gas flows [1].

This paper is not intended to describe in detail or evaluate the two systems; it merely revisits the fundamental characteristics and function of the two tubes, particularly in reference to the tube within a tube design.

## Anesthesia circle circuit (1930)

Since Brian C. Sword introduced the circle (CO_2_ absorption) system in the 1930s [2], the system with 2 separate breathing tubes became the standard anesthetic circle circuit in North America and in most of the countries which followed the American anesthesia technique. Numerous variants of the circle system have been suggested, depending on the site of fresh gas entry, relative positions of the uni-directional valves, the reservoir bag, and the carbon dioxide absorber [3]. However, their basic structure and, in particular, their physical configuration had not been changed: The circle system requires an apparatus which is constructed, so that the inspiratory and expiratory gas flow runs in the same direction (i.e., circular manner). This requires a separation by means of uni-directional valves and two separate tubes, one for inspiration and one for expiration connected by a “Y-piece”, which, in turn, connects to the patient via a mask, endotracheal tube (ETT), or a laryngeal mask (LMA). Thus, the main component which connects the patient’s airway to the anesthesia machine is a pair of tubes.

**Fig. 1:**
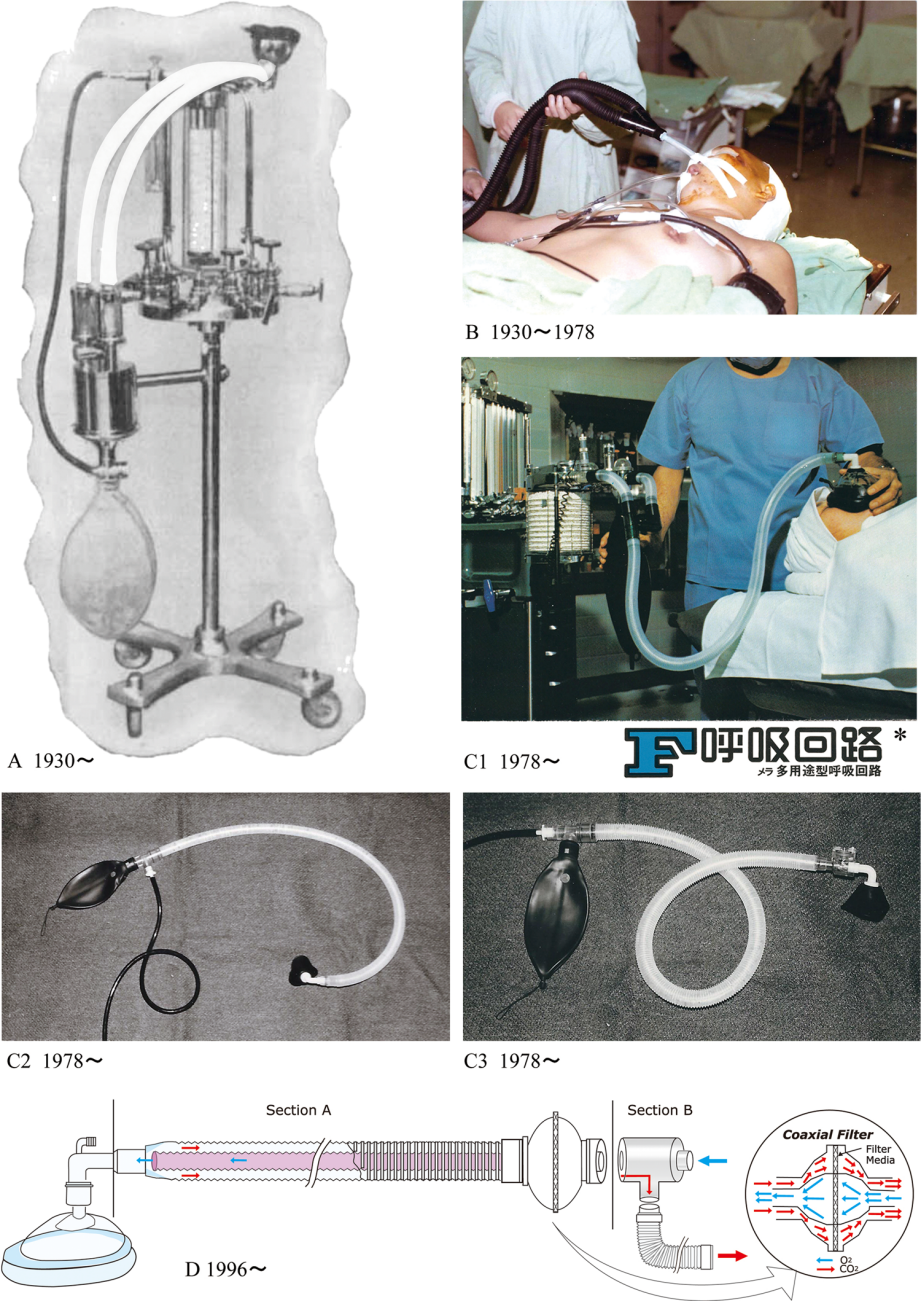
The F Breathing Circuit. **a** The first two-limbed circle circuit reported by Brian C. Sword in 1930 (from Ref. [2]). **b** Conventional two-limbed circuit made of black-corrugated rubber tubes used in Japan until 1978. **c1** The first plastic F Breathing Circuit developed in Japan in 1978 (used as anesthetic circle circuit). * (F Breathing Circuit—MERA multipurpose breathing circuit). **c2** The F Breathing Circuit used as Mapleson-type circuit: partial rebreathing circuit. **c3** The F Breathing Circuit used as a non-rebreathing circuit which includes a non-rebreathing valve at the patient end. **d** The F Breathing Circuit Section A (respiratory limb: inspiratory and expiratory tubes), and Section B (manifold) are constructed separately. Section B is set to the anesthesia machine in a permanent or semi-permanent manner; the bidirectional (coaxial) filter is securely attached to the respiratory limb; the whole component (Section A) can be attachable/detachable from the manifold (Section B) in one connection allowing easy set-up

Despite its physical inconvenience (two separate tubes occluding and creating an obstacle around the face) and other limitations (e.g., cold and dry inspiratory gases), the conventional configuration of the circle system, which uses two separate limbs, was the only circuit available since 1930 (Fig. 1). It took almost 50 years until a viable, safe, single-limb circle circuit became available to the health practitioner [4].

## Mapleson anesthetic system (1954)

In 1954, Mapleson classified five different semi-closed rebreathing anesthetic systems, and designated them as A–E systems; the F designation was later added [1] and refers to the Jackson–Rees circuit. The Mapleson A and D were modified by Lack and Bain, respectively, by placing one tube within the other tube. Bain placed the fresh gas inflow tube within the outer, rebreathing tube [5], whereas the outer tube led the fresh gas flow in the Lack configuration [6].

## The Bain circuit: a coaxial modification of the Mapleson D type circuit (1972)

The well-known coaxial breathing circuit was introduced by Bain and Spoerel in 1972 [5]. However, the Bain circuit was not intended for use in a circle system. The Bain circuit was designed and intended to function as a Mapleson D type circuit: the narrow inner tube mainly functions as a “fresh gas inflowing tube” [5], rather than a respiratory tube. This important characteristic has been pointed out as a significant drawback of the system in several reports. Nott observed that the inner tube is too narrow and insufficient for tidal inspiration, and that “inspired gases come mainly from the (outer) expiratory side of the system and rebreathing is possible” [7]. Other reports point out that the Bain circuit is unusable in a circle system and potentially hazardous [8–10]. Thus, although the Bain circuit initially appeared to offer significant advantages due to its compactness, its use has been limited. Cooper states that, as of 2014, “in the UK, although it is often used in the induction room, it is rarely used in the operating room” [11].

## The F Breathing Circuit: a universal single-limb breathing circuit (1978)

The first universal single-limb breathing circuit was introduced by Fukunaga in 1978 (Japanese Patent No. 1,146,290 filed on May 17, 1978; US Patent No. 4,265,235, filed on May 5, 1981 and based on the Japanese patent; the first clinical report was published in 1978 [4]). The circuit consists of a tube within a tube single-limb circuit that can be used in the circle anesthesia system as well as the Mapleson-type system. Specifically, the circuit has the following characteristics:

- Compact, simple, and light in weight.
- Either tube can provide streamlined inspiratory and expiratory flows in spontaneous, assisted, and controlled ventilation (an essential consideration for a safe breathing circuit).
- When connected in a preferred mode, the outer tube’s expired gases pass through the uni-directional valves and the CO_2_ absorber which flows into the inner, inspiratory tube in one direction and then to the patient (an essential consideration for a circle anesthetic system which provides warmer and more humid inspired gases).
- The inner tube is freely disposed at the patient end (i.e., the inner tube and the outer tube do not need to be anchored at both ends of the circuit, or be maintained in a strictly coaxial or concentric disposition, for example, by stays or spacer means). The freely floating inner, inspiratory tube at the patient end can prevent the potential hazard of inner tube disconnection at the machine end or eliminate breathing obstruction caused by kinking, bending or torsion of the inner, inspiratory tube, so that it will prevent excessive hypercarbia or hypoxia and thus provide a safe circuit.
- Provides simplified construction, which made it feasible to manufacture the circuit more economically.
- Can be used interchangeably as a circle anesthetic system, as a Mapleson type as well as a non-rebreathing circuit (e.g., for ICU ventilation). Thus, the circuit is a universal-type circuit.

The F Breathing Circuit expanded the scope and usage of the single-limb circuit to encompass both the circle system and the Mapleson system in one single system/circuit. Hence, the F Circuit can be universally used; it can safely function in a variety of breathing modalities [4, 12]; it can be connected to an anesthesia machine with or without a carbon dioxide absorber, or be connected to an ICU ventilator. Furthermore, the circuit can be used as a transport circuit in pediatric and adult cases. Because of its versatility and multipurpose functionality, the F Breathing Circuit was described as a “truly universal anesthetic breathing system” [13]. Safety, simplicity, and reliability were particularly emphasized in the development of the circuit. Hence, the inner and outer tubes were made to reliably enable inspiration and expiration in spontaneous and controlled ventilation at all times—even if the tubes are connected in a reversed manner (outer tube to inspire and inner tube to expire gases), the circuit will not be hazardous. The former mode is recommended because of its advantages (countercurrent heat exchange between the cold fresh gas, inspired gas, and the warmer expired streams) [14, 15]. The F Circuit subsumes both the circle circuit and Mapleson circuit types. The same circuit can be seamlessly used throughout different stages and modalities during the course of anesthesia: (a) for induction of anesthesia (in the induction room [in places where induction of anesthesia is performed in a separate location from the operating room]: use as the Bain circuit), (b) for induction and maintenance of anesthesia (in the operating room: use in the circle system), (c) for recovery of anesthesia (in the recovery room: use as the T-piece), (d) for ventilating a patient in need of oxygenation (in the ICU), or (e) for transporting the patient while ventilating (use as a Jackson–Rees circuit or in lieu of the Ambu bag device).

## Development of the F Breathing Circuit (1978, 1992)

In reference to the F Breathing Circuit, a prototype made of plastic tubing comprising all the required characteristic mentioned above could be accomplished in a feasible, viable manner; The circuit was tested in the laboratory with a model lung and by volunteers. Thereafter, a clinical study was designed and evaluation of the circuit was performed. The circuit was approved for commercialization by the government agency in Japan (1978), and subsequently, development and commercialization were undertaken by Senko Medical Instrument Mfg Ltd (MERA), Tokyo, Japan.

The circuit was first identified as “Fukunaga Breathing Circuit”. The name was then abbreviated to “F Kairo,” “F Circuit,” “F Breathing Circuit,” “F Universal Circuit”, and “Mera F Circuit” (Fig. 1).

Over a decade later (1992), after the circuit had been widely used in Japan, the technology was further evolved and commercialized in the US and worldwide by King Systems Corporation (Ambu) of Indiana [16].

Several modifications, improvements, and expanded usages followed the first commercial circuit. One significant improvement is the provision of a separate manifold which is attached to the inspiratory and expiratory ports of the anesthesia machine, respectively (Fig. 1d). The manifold can be set in a permanent or semi-permanent manner. The single-limb circuit can be connected to the manifold with one motion, one connection, and greatly simplifying set-up. The connection can be done via a coaxial filter which is attached to the coaxial connector at the machine end of the hoses. The coaxial filter design distributes flow into dedicated inspiratory and expiratory quadrants. This provides filtration of both inspiratory and expiratory ends, thereby guaranteeing protection of both: the anesthesia machine and the patient, while eliminating the need of two filters and/or need for disposing the manifold. This simplifies the usage and saves set-up time, since the user does not need to make multiple connections or use two filters. Another improvement to the F Breathing Circuit was the use of two flexi tubes (accordion-like pleated tubes) which could be expanded and contracted simultaneously in a synchronized manner while maintaining the desired curvature, position, and length of the single-limb circuit. These later improvements added great flexibility and convenience to the unilimb circuit, and led to widespread, worldwide use of the USLBC under the trade names “Universal F,” “Universal F2,” “Ped F2,” and “KingUniversal F.”

The F Breathing Circuit has been used in millions of patients and veterinary cases worldwide, and although the present circuit can be readily used in a variety of modalities, the single-limb universal breathing circuit currently appears to be mainly associated with its use as a circle circuit. However, it should be kept in mind that a single F Circuit can function in different modes, minimizing the need for multiple circuits and devices—this important fact should be kept in mind as environmental and economic pressures necessitate the more efficient usage of medical devices.
